# Enhanced Hybridization Selectivity Using Structured GammaPNA Probes

**DOI:** 10.3390/molecules25040970

**Published:** 2020-02-21

**Authors:** Taylor D. Canady, April S. Berlyoung, Joe A. Martinez, Cole Emanuelson, Cheryl A. Telmer, Marcel P. Bruchez, Bruce A. Armitage

**Affiliations:** 1 Department of Chemistry and Center for Nucleic Acids Science and Technology, Carnegie Mellon University, 4400 Fifth Avenue, Pittsburgh, PA 15213-3890, USA; canmatics@gmail.com (T.D.C.); april.berlyoung@gmail.com (A.S.B.); joe.martinez.jam@gmail.com (J.A.M.); emanuelsonc@gmail.com (C.E.); bruchez@cmu.edu (M.P.B.); 2 Department of Biological Sciences, Carnegie Mellon University, 4400 Fifth Avenue, Pittsburgh, PA 15213-3890, USA; ctelmer@cmu.edu

**Keywords:** γPNA, antisense, hybridization, selectivity

## Abstract

High affinity nucleic acid analogues such as gammaPNA (γPNA) are capable of invading stable secondary and tertiary structures in DNA and RNA targets but are susceptible to off-target binding to mismatch-containing sequences. We introduced a hairpin secondary structure into a γPNA oligomer to enhance hybridization selectivity compared with a hairpin-free analogue. The hairpin structure features a five base PNA mask that covers the proximal five bases of the γPNA probe, leaving an additional five γPNA bases available as a toehold for target hybridization. Surface plasmon resonance experiments demonstrated that the hairpin probe exhibited slower on-rates and faster off-rates (i.e., lower affinity) compared with the linear probe but improved single mismatch discrimination by up to a factor of five, due primarily to slower on-rates for mismatch vs. perfect match targets. The ability to discriminate against single mismatches was also determined in a cell-free mRNA translation assay using a luciferase reporter gene, where the hairpin probe was two-fold more selective than the linear probe. These results validate the hairpin design and present a generalizable approach to improving hybridization selectivity.

## 1. Introduction

High affinity recognition and accurate discrimination of nucleic acids is the foundation for many biotechnology tools and biomedical applications ranging from PCR-based diagnostics and fluorescence in situ hybridization (FISH) to antisense blocking of translation and genome editing. Nevertheless, the enduring challenge of technologies that rely on nucleic acid hybridization is the trade-off between affinity and selectivity [1]. The affinity of an oligonucleotide probe for its DNA or RNA target can be fine-tuned by adjusting the length and/or the nucleobase composition. On the other hand, optimizing the selectivity of a probe for its target is much more difficult due to the demand for discriminating single mismatches. Making the probe short so that a mismatch prevents hybridization at the experimental temperature raises the likelihood that other perfectly matched sequences will be present elsewhere in the genome/transcriptome, leading to off-target hybridization. Making the probe long in order to find a unique sequence also enables off-target hybridization due to the modest destabilizing effect of a single mismatch in a high-affinity probe.

The quest to block off-target hybridization has led to many innovative approaches that vary with the application. Of relevance to the work we report here is the strategy of intentionally building a secondary structure into the probe [2,3,4]. Thus, intramolecular base pairing within the probe competes with intermolecular base pairing to the target. This naturally decreases the affinity of the structured probe for its target, relative to a corresponding unstructured probe, but it increases the sensitivity to a single mismatch.

**Scheme 1:**
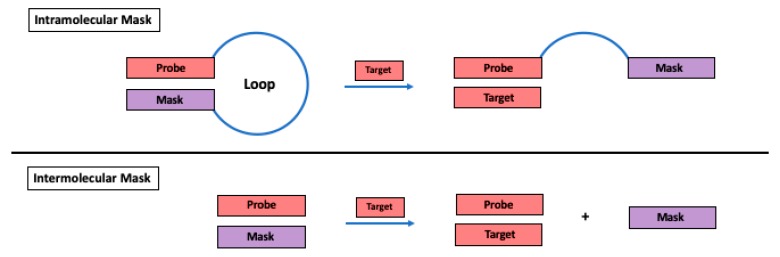
Use of a masking oligonucleotide to improve selectivity of hybridization probes.

The simplest type of nucleic acid secondary structure is the hairpin, in which two remote complementary sequences form a stem through Watson–Crick base pairing, extruding the intervening nucleotides into a loop. One side of the stem, i.e., the probe, is complementary to the target, while the other side of the stem serves as the “mask”, which provides the competition (Scheme 1). The ability of a hairpin secondary structure to improve hybridization selectivity was first reported in the context of fluorescent molecular beacons [2,3] and has since been extended to other types of hybridization probes [4]. In addition, intermolecular masking of a probe through hybridization to a competing oligonucleotide in order to improve selectivity has also been reported (Scheme 1) [5,6].

As chemists have developed new generations of synthetic oligonucleotides, significant affinity improvements have been realized. Peptide nucleic acid (PNA) is one such example [7,8], where the high affinity and the biochemical stability have led to numerous applications, including PCR-clamping [9] and bacterial genotyping [10], with even higher affinity reported for a second-generation PNA known as gammaPNA (γPNA) [11,12], which features a helix-inducing substituent at the gamma carbon of the PNA backbone. A 10mer γPNA binds to its complementary DNA and RNA targets with mid-fM K_d_ values [12]. The high affinity of γPNA allows it to invade genomic DNA in order to bind its target site, leading to applications in in vivo gene editing [13,14] and telomere FISH [15,16].

While high affinity is essential for applications requiring invasion of genomic DNA, use of γPNA in other contexts where the target site is more accessible (e.g., antisense) is susceptible to off-target effects due to the tolerance for single mismatches. This report describes our efforts to use a hairpin secondary structure to improve the selectivity of a γPNA probe.

## 2. Results

### 2.1. Hairpin Design and Characterization

**Scheme 2:**
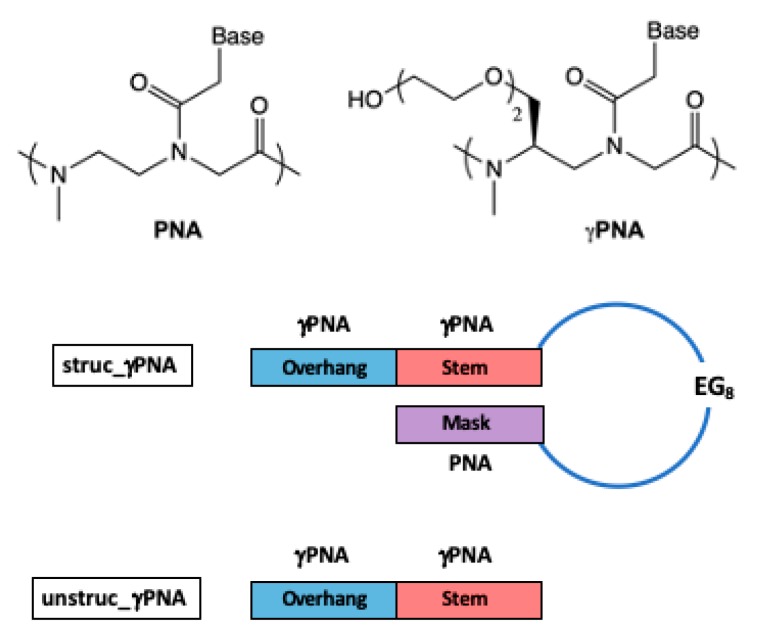
Chemical structures of PNA and γPNA and design of structured and unstructured γPNA probes.

To test the impact of secondary structure on γPNA hybridization selectivity, we designed struc_γPNA, which consists of a 10 nucleotide (nt) target-recognition domain composed of γPNA monomers, an octaethylene glycol (EG_8_) linker, and a 5 nt PNA mask complementary to the last 5 nt of the target-recognition domain (Table 1). The probe should fold into a hairpin structure [17] with a five base pair (bp) γPNA-PNA stem, an EG_8_ loop, and a 5 nt γPNA overhang, or “toehold”, where hybridization to a target can be nucleated (Scheme 2) [18]. Using PNA rather than γPNA as the mask should decrease the stability of the stem [19], thereby increasing the likelihood that binding to a target nucleic acid will result in opening of the stem to form a stable duplex. Designing the probe to have an overhang should minimize any penalty in hybridization kinetics compared with a traditional molecular beacon design, where the stem is fully base-paired. The unstructured probe (unstruc_γPNA) consisting solely of the 10 nt γPNA target-recognition domain was also synthesized for comparison.

**Figure 1:**
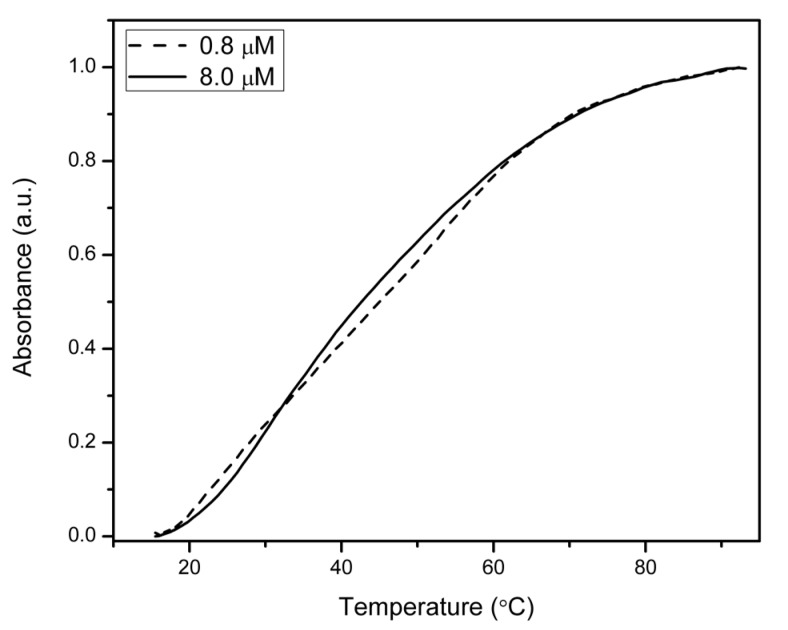
UV melting transition observed at 275 nm for struc_γPNA is independent of strand concentration over a 10-fold range. Buffer contained 10 mM Tris-HCl (pH = 7.4), 100 mM NaCl, 0.1 mM Na_2_EDTA.

Intramolecular folding of struc_γPNA was confirmed by UV melting studies, in which a melting transition was observed at T_m_ = 40 °C at both 0.8 and 8.0 μM strand concentrations (Figure 1). The lack of a concentration dependence for the transition is consistent with formation of an intramolecular hairpin as opposed to an intermolecular duplex. Although γPNAs generally exhibit hyperchromic transitions near 40 °C due to melting of their intrinsic helical structure (even when their sequences do not allow hairpin formation), kinetic data presented below are consistent with hairpin formation by struc_γPNA. For comparison, we recently reported a similar hairpin consisting of a 4 bp γPNA-γPNA stem and a peptide loop, which melted at 76 °C [20].

### 2.2. Hybridization Selectivity Analyzed by SPR

We used surface plasmon resonance (SPR) experiments to characterize hybridization of the structured and the unstructured γPNA probes to DNA targets that were either perfectly matched to the 10mer recognition domain of the probes (PM) or would form a single mismatch to various positions within either the stem or the overhang region of the structured probe. For each experiment, a biotinylated version of the DNA target was immobilized on a streptavidin-coated chip, and a solution of either struc_γPNA or unstruc_γPNA at various concentrations was flowed over the chip. The sequences of the DNA targets are given in Table 1 and are named so as to indicate the location of the mismatch in either the stem (S) or the overhang (O), and the specific position and the mutation (e.g., MM-7T indicates the base at position seven is a T).

**Figure 2:**
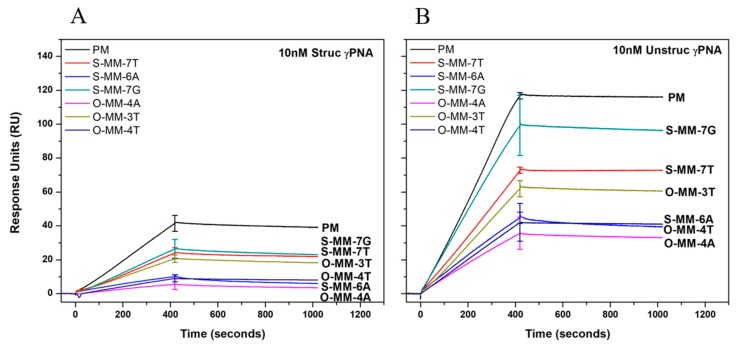
Surface plasmon resonance (SPR) sensorgrams for 10 nM struc_γPNA (**A**) and unstruc_γPNA (**B**). DNA target sequences are given in Table 1. All data are plotted as *n* = 3 average ± SD.

Figure 2 shows SPR sensorgrams for hybridization of 10 nM struc_γPNA (A) and unstruc_γPNA (B) to the various DNA targets. It is immediately apparent that the on-rate for the structured probe is ca three-fold lower than for the unstructured probe. Two factors likely contribute to this difference. First, the unstructured probe can nucleate via any subset of its 10 nucleotides, which are all accessible to the target, whereas the PNA mask on the structured probe blocks access to five of the nucleotides. Second, even if nucleation between struc_γPNA and a DNA target does occur via the toehold, the time required to open the hairpin and form additional base pairs to stabilize the duplex could allow the probe to dissociate from the immobilized target.

There is also a smaller but measurable difference in off-rate for the two probes with their perfect match targets. The dissociation phase of the experiment, where buffer rather than probe is flowing over the surface of the chip, begins at ca. 420 s. The unstructured probe shows very little dissociation from the PM target, consistent with prior reports of γPNA kinetics [12,21]. In contrast, the structured probe clearly begins to dissociate from the chip surface as soon as the buffer begins flowing over the surface; Figure S1 (Supplementary Materials) shows an overlay of the two dissociation curves. The difference likely arises because the struc_γPNA can reform the hairpin structure as it dissociates from the DNA target, slowing its re-association and leading to net loss from the chip surface.

**Figure 3:**
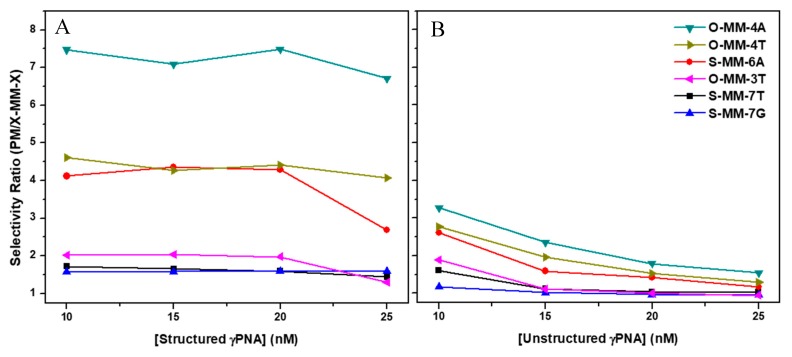
Selectivity ratio of (**A**) struc_γPNA and (**B**) unstruc_γPNA as determined by dividing the association max (max response units (RU) in Figure 1 and Figure 2) of the perfect match to each respective target mutant association max.

Compared to the perfect match target, all mismatch targets showed slower hybridization for both the structured and the unstructured probes. In order to compare the selectivities of the two probes, we divided the maximum response units (determined at t = 420 s) for the perfect match targets by those for each mismatch target. The resulting selectivity ratios (SR) are plotted in Figure 3 for each of the γPNA concentrations tested. The improved selectivity for the structured probe is evident, with higher SR values for each target. Moreover, the SR is invariant in the range of 10–20 nM for struc_γPNA but decreases nearly two-fold for unstruc_γPNA. Overall, these results demonstrate that the hairpin secondary structure of struc_γPNA leads to significant improvement in mismatch discrimination.

It is clear from the SPR data that the position of the mismatch has a significant effect on the hybridization selectivity, even though the order of selectivity is not dependent on whether the probe is structured. Since we only studied a subset of all possible mismatches and locations, it is interesting to see that the most strongly discriminated mismatches are those that are closer to the middle of the probe (O-MM_4A, O-MM-4T, and S-MM-6A). This is consistent with prior literature demonstrating the greater destabilizing effects of internal mismatches as opposed to terminal mismatches, where fraying of the base pairs weakens discrimination of noncomplementary nucleotides [22].

For the structured probe, the nature of the mismatch at position four has a significant effect on selectivity; the SR for O-MM-4A (A-G mismatch) is 70% higher than for O-MM-4T (T-G mismatch), likely due to the ability of the latter target to form a more stable G-T wobble pair with the γPNA probe [22]. In contrast, the unstructured probe only discriminates the G-A mismatch by 17% over the G-T mismatch. The nature of the mismatch at position 7 (T-T vs. G-T) does not significantly impact selectivity, although the SR < 2 for these targets leaves little opportunity for selectivity differences to emerge.

### 2.3. UV Melting Analysis of Select γPNA-DNA Duplexes

The improved selectivity of the structured γPNA probe was evident in the kinetics experiments presented above. We next characterized the thermal stability of the duplexes formed between the γPNA probes and three DNA targets, namely the perfect match and the O-MM-4X targets, where kinetic discrimination was highest. Cooperative, reversible melting transitions were observed in all cases; melting curves can be found in the Supporting Information. The melting temperatures (T_m_) are collected in Table 2.

The data indicate that the hairpin conformation of struc_γPNA does not have a detrimental effect on DNA hybridization. In fact, the T_m_ values are 2–4 °C higher for the structured versus the unstructured probe. This implies that the overhanging nucleotides present after hybridization of struc_γPNA provide added stability. While this phenomenon was reported previously for PNA [23], the EG_8_ linker separating the PNA mask from the γPNA target-recognition domain in the present case would be expected to minimize interactions between the two domains after hybridization. Of greater interest is the fact that the effect of mismatches on melting (ΔT_m_) is about 2 °C lower for the structured probe. The lack of any improvement in selectivity in these experiments can be attributed to the fact that the hairpin secondary structure is significantly destabilized at the temperatures where the mismatched duplexes melt. Since the hairpin cannot re-fold after melting, no effect on mismatch discrimination is observed, in contrast to the kinetic discrimination observed at 25 °C in the SPR experiments described above.

### 2.4. Antisense Effects in a Luciferase Reporter Assay

**Figure 4:**
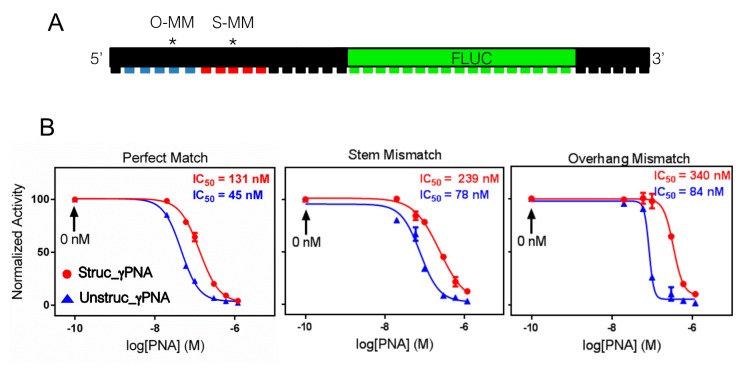
(**A**) Design of firefly luciferase (Fluc) mRNA targets for antisense experiments with unstructured and structured γPNA probes performed in rabbit reticulocyte lysate. Mismatch sites are indicated with (*). (**B**) Dose-response curves determined after 1 h annealing at 37 °C.

Both γPNA probes are complementary to a 10 nucleotide site at the 5′-UTR terminus of a firefly luciferase (Fluc) mRNA, which was shown previously to be a potent antisense target site for γPNA [24]. We also constructed mutant Fluc mRNAs bearing mismatches within either the stem (5′-AGACCUAGC-3′) or the overhang (5′-AGAACCAAGC-3′) regions of the target-recognition site (Figure 4A). These correspond to DNA probes S-MM-7T and O-MM-4A from the earlier experiments, where strong kinetic discrimination was observed for the overhang mismatch but not the stem mismatch (Figure 3).

To compare the antisense activity of the γPNAs, we annealed various concentrations of either probe with freshly transcribed mRNA for one hour at 37 °C before adding to rabbit reticulocyte lysate to start translation. Dose-response curves shown in Figure 4B reveal nearly three-fold higher potency for the unstructured probe against the perfect match mRNA (IC_50_ = 45 nM vs. 131 nM). Since these experiments were done at a temperature below the T_m_ for unfolding of the hairpin, struc_γPNA would be expected to be less potent. When a U-T mismatch was present in the stem domain, the IC_50_ values for the two probes increased by roughly the same amount, i.e., the unstructured probe remained three-fold more potent. However, when an A-G mismatch was present in the overhang domain, the IC_50_ value for the structured probe increased by 42%, while that for the unstructured probe increased only 7%, leading to a four-fold difference in potency. Thus, the mismatch discrimination effects observed for O-MM-4A and S-MM-7T by SPR and luciferase reporter assay are qualitatively consistent.

**Figure 5:**
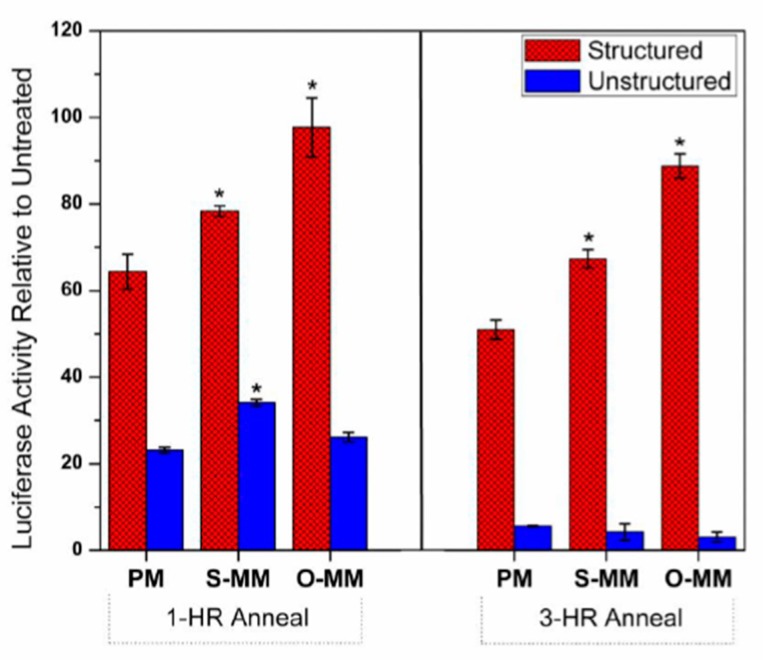
Effect of annealing time on antisense target discrimination. Samples contained 10 nM mRNA and 100 nM γPNA. Fluc activity was compared to control samples lacking either γPNA probe and are expressed as a percentage of activity on the *y*-axis. At a 1 h annealing time (left panel), the struc_γPNA demonstrated a statistically significant (* = *p <* 0.05) difference in luciferase inhibition between the perfect match (PM) and both mismatch targets, whereas the unstruc_γPNA only showed a significant difference between the PM and S-MM-7U. At a 3 h annealing time (right panel), there was no inhibition difference (*p* > 0.05) amongst the three targets when using the unstruc_γPNA, whereas the struc_γPNA retained discrimination of the mismatched targets. All data shown are presented as an average of *n =* 3 ± SD.

We repeated these experiments at a single γPNA concentration (100 nM) but with an extended annealing time of 3 h to determine whether the effects shown in Figure 4 were due to kinetic differences in probe hybridization. The results are shown in Figure 5, where the relative luciferase activity (i.e., the ratio of luciferase signal in the presence and the absence of the γPNA) is plotted for both probes and all three targets after 1 or 3 h annealing. Thus, a higher value corresponds to a weaker antisense effect. For struc_γPNA, extended annealing did not alter the selectivity of the probe. Slightly greater knockdown of luciferase was observed. Interestingly, unstruc_γPNA exhibited much larger effects due to prolonged annealing. Specifically, greater knockdown was observed, but the selectivity was lost. It is unclear why the unstructured probe appeared to lose selectivity at the longer annealing time. However, the important finding of this experiment is that the ability of the structured probe to discriminate between perfect and mismatched targets is due to thermodynamic rather than kinetic differences.

## 3. Discussion

High affinity oligonucleotides such as γPNA possess the ability to overcome target secondary and tertiary structures that impose kinetic and thermodynamic obstacles to hybridization. Even genomic DNA can be successfully hybridized by γPNA, as evidenced by recent reports of in vivo [13] and in utero [14] gene editing induced by strand-invading γPNAs. However, the capacity to target highly structured DNA or RNA comes with a substantial risk, namely tolerance of single- or even multiple-mismatch sites, potentially leading to off-target hybridization. In the best case, this would diminish the potency of on-target hybridization while, in the worst case, it would lead to deleterious side effects. The results described above illustrate a generalizable approach to improving the selectivity of γPNAs.

The SPR results demonstrate the impact of the hairpin design on hybridization selectivity. Compared to the unstructured analogue, struc_γPNA showed ca. three-fold slower hybridization kinetics, as expected due to needing to unfold the hairpin structure in order to fully hybridize to the DNA target. There was also a slight increase in off-rate, presumably due to the ability of struc_γPNA to refold into a hairpin upon dissociation. Slower binding and faster release translate into decreased overall affinity. This was not reflected in the melting data due to the fact that the hairpin structure melts at a lower temperature than even the mismatched duplexes. Although the overall affinity was lower, the selectivity of struc_γPNA was markedly improved over that of unstruc_γPNA for each of the single mismatches tested (Figure 3). Moreover, we observed that the selectivity of the hairpin probe was relatively insensitive to γPNA concentration, whereas the linear probe’s selectivity decreased progressively as its concentration increased. For example, struc_γPNA was 2.2-fold more selective at 10 nM concentration, but this rose to 4.5-fold at 25 nM probe.

The hairpin probe also exhibited higher selectivity in cell-free antisense experiments after both 1 and 3 h annealing times. The selectivity of struc_γPNA was similar for both annealing times, whereas unstruc_γPNA’s selectivity was lost at the longer annealing time. Evidently, longer time allows the unstructured probe to hybridize to the mismatch sites, leading to inhibition of translation. On the other hand, the kinetic barrier imposed by the hairpin structure presented a sufficiently strong barrier to hybridization to the off-target sites to allow it to retain its selectivity even after extended annealing.

Both probes exhibited greater antisense effects after longer incubation, but the improved potency was much more apparent for the unstructured γPNA, suggesting a greater kinetic barrier to hybridization by this probe compared with struc_γPNA. This is surprising, given the faster hybridization and the slower dissociation of this probe in the SPR experiments. One possible explanation for this result is initial spurious hybridization of unstruc_γPNA to off-target sites elsewhere in the luciferase mRNA before binding to the higher affinity perfect match site in the 5′-UTR. Such sites might be more accessible and/or abundant than the target site, allowing faster hybridization, yet their lower affinity would minimize any potential antisense effects. In contrast, the structured probe would have slower association at the off-target sites, leading to an antisense effect that was nearly maximized even after only 1 h of incubation.

The improved mismatch discrimination of struc_γPNA validates the hairpin design, but much more needs to be done to optimize the approach. In particular, the length and the affinity of the mask domain need to be studied in detail. One would expect the selectivity to improve with the stability of the hairpin, but the cost in potency could be substantial. Moreover, moving the mask to different positions along the probe could lead to significant differences in both selectivity and potency. Finally, analyzing probe selectivity through both biophysical and biochemical methods is important since real-world performance in an application can be affected by factors beyond hybridization kinetics and affinity.

## 4. Materials and Methods

### 4.1. γPNA/DNA Oligomers

All γPNA oligomers reported here were purchased from PNA Innovations Inc and gave satisfactory HPLC and MS data. This vendor is no longer in business. Concentrations of struc_γPNA (ε_260_ = 146,700 M^−1^ cm^−1^) and unstruc_γPNA (ε_260_ = 94,400 M^−1^ cm^−1^) were determined by UV-vis absorption on a Cary 300 spectrophotometer (Agilent, Santa Clara, CA, USA). Sequences of all biotinylated target DNA oligonucleotides used in SPR direct binding experiments are also given below. DNA oligonucleotides were ordered from Integrated DNA Technologies (idtdna.com, Coralville, IA, USA).

### 4.2. Surface Plasmon Resonance (SPR)

SPR experiments were performed using a Biacore T100 instrument (GE Healthcare, Marlborough, MA, USA) and four-channel carboxymethyl dextran matrix sensor chips. These commercially available CM5 chips (GE Healthcare) were further functionalized with streptavidin (approximately 5000 RUs) via NHS-EDC coupling. The 5′ biotinylated DNA targets (all oligonucleotide DNA targets used are shown in the Table 3) were individually immobilized (approximately 120 RUs) to the streptavidin labeled surface, and chips were finalized for PNA injections by priming five times with buffer. The buffer used for chip preparation and all subsequent SPR experiments was 10 mM HEPES pH 7.4, 100 mM NaCl, 3 mM Na_2_EDTA, and 0.005% *v*/*v* polysorbate 20 (HBS-EP Buffer).

Direct binding experiments were conducted in triplicate (sensorgrams provided are an average of all three experiments with a standard deviation shown at 418 s) as well as background subtracted for streptavidin and buffer backgrounds. Various concentrations (10, 15, 20, or 25 nM) of structured or unstructured γPNA were injected over the prepared sensor chip for 400 s (flow rate = 30 μL/min). This was followed by a dissociation cycle via buffer injection for 600 s (flow rate = 30 μL/min). Finally, a regeneration cycle was conducted to wash any residual γPNA from the sensor chip (30 s injection of 1 M NaCl, 10 mM NaOH flow rate = 50 μL/min), followed by a buffer injection to re-establish a baseline for subsequent injections (150 s injection of buffer flow rate = 30 μL/min).

Hybridization on rates were calculated using the slopes of the raw sensorgrams between 80–100 s (Equation (1)).

(1) $$\mathrm{On}\;\mathrm{Rate}=\frac{{\mathrm{RU}}_{80\;s}-{\mathrm{RU}}_{100\;s}}{20\;s}$$

On rate ratios were calculated by dividing the PM on rate by each mismatch (Equation (2)).

(2) $$\mathrm{On}\;\mathrm{Rate}\;\mathrm{Ratio}=\frac{{\mathrm{PM}}_{\mathrm{On}\;\mathrm{Rate}}}{{\mathrm{MM}}_{\mathrm{On}\;\mathrm{Rate}}}$$

Selectivity ratios were calculated for all mismatch DNAs using the maximum at 418 s. Max RUs of the PM were divided between that of the mismatches in order to compare the penalty each mismatch imposed on γPNA binding (Equation (3)).

(3) $$\mathrm{Selectivity}\;\mathrm{Ratio}=\frac{{\mathrm{PM}}_{\mathrm{MAX}}}{{\mathrm{MM}}_{\mathrm{MAX}}}$$

### 4.3. UV Melting

Samples containing 2 μM each of DNA target and γPNA probe were mixed in buffer containing 10 mM Tris-HCl (pH = 7.4), 100 mM NaCl, and 0.1 mM Na_2_EDTA and placed in 1 mL, 1 cm pathlength quartz cuvettes. Samples were heated to 90 °C for 5 min before cooling to 15 °C at a rate of 1 °C/min, collecting absorbance values at 260 nm at 1.0 °C intervals. Samples were then heated back to 90 °C at the same rate and data collection interval. No hysteresis was observed between heating and cooling curves; heating curves are shown in the text.

### 4.4. Luciferase Assays

Mutated Fluc template production and in vitro transcription were performed. A previously cloned firefly luciferase template (T7 promoter) was used as the “perfect match” template (Fluc-PM) and used to create the additional O-MM-4 (Fluc-O-MM) and S-MM-7 (Fluc-S-MM) firefly templates. To create the mutant subclones, Fluc-PM was digested at a PvuII (upstream of T7 promoter) and HindIII (downstream of T7 promoter) cut site. The digested Fluc vector was then purified using agarose gel electrophoresis (0.4%). The O-MM-4 and the S-MM-7 forward and reverse sequences were ordered from Integrated DNA Technologies (idtdna.com) and contained the PvuII and HindIII overhangs. The O-MM-4 and the S-MM-7 oligonucleotides were pre-annealed into duplex formation prior to T4-ligation.

***O-MM-4A** insert sequences*:

5′-CTG GCT TAT CGA AAT TAA TAC GAC TCA CTA TAG GGA GAA CCA

5′-AGC TTG GTT CTC CCT ATA GTG AGT CGT ATT AAT TTC GAT AAG CCA G

***S-MM-7U** insert sequences*:

5′-CTG GCT TAT CGA AAT TAA TAC GAC TCA CTA TAG GGA GAC CCT

5′-AGC TAG GGT CTC CCT ATA GTG AGT CGT ATT AAT TTC GAT AAG CCA G

After ligation, the plasmid was transfected (Mach1/T1 *Escherichia coli*) and plated (plasmid confers ampicillin resistance) overnight. The resultant colonies were selected and sent for sequencing for verification.

PCR amplification of the firefly luciferase plasmid was then performed. The firefly plasmids were PCR amplified using the NEB PCR Protocol for Phusion High-Fidelity DNA Polymerase (cycled 35 times, PCR program 98 °C, 2 min; 98 °C, 10 s; 45 °C, 15 s; 72 °C, 2 min; 72 °C, 1 min; hold at 4 °C).

Primer design: T7 transcription site 5′-TACGACTCACTATAGGG

poly A tail site: 5′-TTTTTTTTTTTTTTTTTTTTTTTTTTTTTT

The products were purified using the Thermo Scientific Gel Extraction Kit protocol (Thermo Scientific, Waltham, MA, USA) and verified using agarose gel electrophoresis (1.8 kB).

### 4.5. Transcription Reaction and Purification

The transcription reaction followed the Thermo Scientific conventional transcription protocol (50 μL final volume) and consistently gave high RNA product yield (~2.5 μM, determined via NanoDrop spectrophotometer, Thermo Fisher, Waltham, MA, USA). The transcription reaction was conducted at 37 °C for 2 h. The transcription products were purified using the GeneJET RNA Cleanup and Concentration Micro Kits (Thermo Scientific, Waltham, MA, USA), and concentration was measured using a NanoDrop spectrophotometer.

The γPNA (struc_γPNA or unstruc_γPNA) and the mRNA were annealed together in the presence of 79 mM potassium chloride (designed to match the K^+^ concentration in the rabbit reticulocyte lysate, RRL Promega) and DEPC-treated water. The RNA concentration for all translation experiments was set at 10 nM in the final translation reaction at a final volume of 15 μL. The probe concentration varied depending on the desired dose. The probe/mRNA was annealed at 37 °C for 1 h.

The translation reaction was conducted using the Luciferase Assay System (E1500, Promega, Madison, WI, USA) (rabbit reticulocyte lysate). The entire 15 μL of annealing solution (above) was mixed into 20 μL lysate. The final γPNA concentration was determined based on the 35 μL final translation reaction volume. The translation reaction was conducted at 30 °C for 1.5 h. Then, 15 μL of lysate solution was mixed into 15 μL of Promega Luciferase Assay Reagent (E1483) and added to a Nunc 96 well plate (flat white, Thermo-Fisher, Waltham, MA, USA). The bioluminescence reading was collected on a TECAN Infinite M1000 plate reader (TECAN, Morrisville, NC, USA).

## Figures and Tables

**Table 1:** GammaPNA (γPNA) probes and DNA targets.

Sequence Name	Sequence ^a,b^	Mismatch
struc_γPNA	H_2_N-K-TCTGGGTTCG-EG_8_-cgaac-H	N/A
unstruc_γPNA	H_2_N-K-TCTGGGTTCG-H	N/A
Perfect Match (PM)	5′-AGACCCAAGC-3′	N/A
Stem Mismatch (S-MM-7T)	5′-AGACCCTAGC-3′	(T-T)
Stem Mismatch (S-MM-6A)	5′-AGACCAAAGC-3′	(A-G)
Stem Mismatch (S-MM-7G)	5′-AGACCCGAGC-3′	(G-T)
Overhang Mismatch (O-MM-4A)	5′-AGAACCAAGC-3′	(A-G)
Overhang Mismatch (O-MM-3T)	5′-AGTCCCAAGC-3′	(T-T)
Overhang Mismatch (O-MM-4T)	5′-AGATCCAAGC-3′	(T-G)

^a^ γPNA upper case, PNA lower case, sequences written C-to-N. (The C-terminus is an amide, hence “H_2_N”). ^b^ Mismatches in DNA targets underlined.

**Table 2:** UV melting temperature (°C) for select γPNA-DNA duplexes.

Target	unstruc_γPNA	struc_γPNA
**PM**	77.6 ± 0.1	80.0 ± 1.4
**O-MM-4A**	54.3 ± 1.3	58.7 ± 0.1
**O-MM-4T**	61.9 ± 0.1	66.0 ± 1.4

**Table 3:** Biotinylated DNA oligonucleotides used for SPR experiments.

DNA Sequence	Sequence ^a^
Perfect Match (PM)	5′-Bt-TTTTTAGACCCAAGC-3′
Stem Mismatch (S-MM-7T)	5′-Bt-TTTTTAGACCCTAGC-3′
Stem Mismatch (S-MM-6A)	5′-Bt-TTTTTAGACCAAAGC-3′
Stem Mismatch (S-MM-7G)	5′-Bt-TTTTTAGACCCGAGC-3′
Overhang Mismatch (O-MM-4A)	5′-Bt-TTTTTAGAACCAAGC-3′
Overhang Mismatch (O-MM-3T)	5′-Bt-TTTTTAGTCCCAAGC-3′
Overhang Mismatch (O-MM-4T)	5′-Bt-TTTTTAGATCCAAGC-3′

^a^ Bt = Biotinylated, mismatches underlined.

## Supplementary Materials

The following are available online. Figure S1: Overlay of SPR sensorgrams for struc_γPNA and unstruc_γPNA with perfect match DNA target showing greater dissociation of struc_γPNA; Figure S2: UV melting curves of unstruc_γPNA with perfect match and single mismatch DNA targets; Figure S3: UV melting curves of struc_γPNA with perfect match and single mismatch DNA targets.
