# Supplementary material for: Enhanced Hybridization Selectivity Using Structured GammaPNA Probes
Source: Molecules. 2020 Feb 21;25(4):970. doi: 10.3390/molecules25040970 (PMC7070858; doi:10.3390/molecules25040970)
Supplement: Supplementary file 1 [file molecules-25-00970-s001.pdf]

SUPPORTING INFORMATION FOR

Enhanced Hybridization Selectivity Using Structured γPNA Probes

Canady, et al

Figure S1. Overlay of SPR sensorgrams for struc_γPNA and unstruc_γPNA with perfect match DNA target showing greater dissociation of struc_γPNA.

Figure S2. UV melting curves of unstruc_γPNA with perfect match and single mismatch DNA targets

Figure S3. UV melting curves of struc_γPNA with perfect match and single mismatch DNA targets

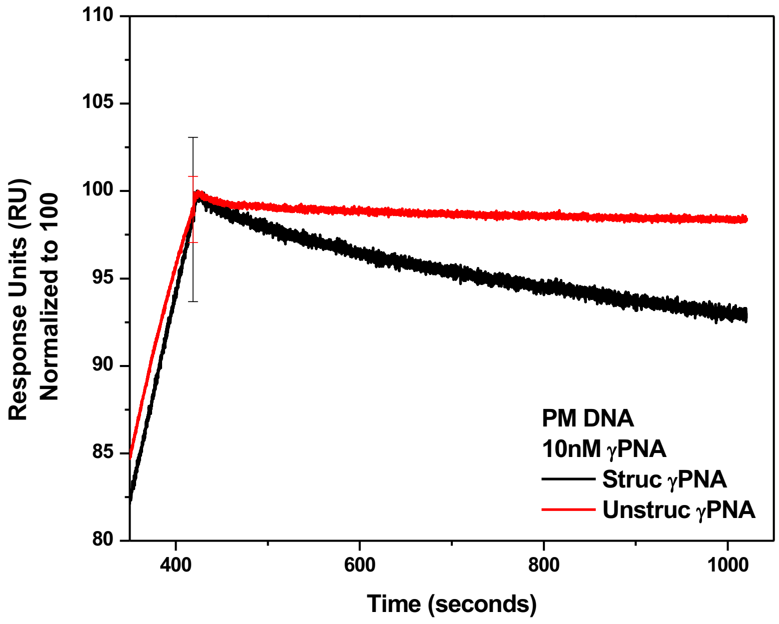

Figure S1. Overlay of SPR sensorgrams showing binding of 10 nM struc_γPNA or unstruc_γPNA to perfect match DNA target. Sensorgrams were scaled to 100% at time where flow changed from γPNA to buffer. Structured probe exhibits significantly greater dissociation from chip.

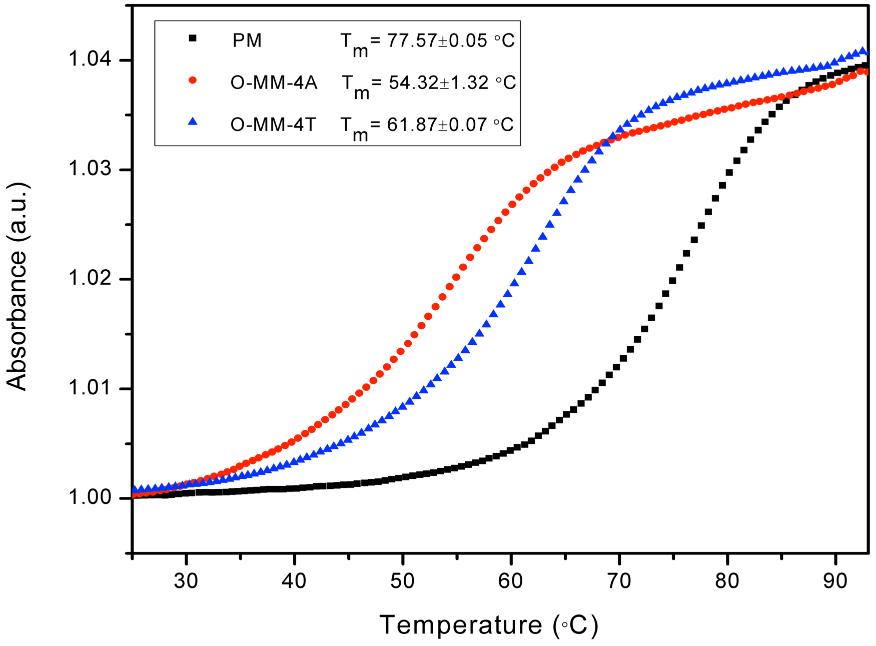

Figure S2. UV-vis melting analysis of unstruc_γPNA with PM, O-MM-4T and O-MM-4A DNA. All samples contained 2 μM DNA and 2 μM unstruc_γPNA in 100 mM NaCl, 10 mM Tris-HCL (pH = 7.4), and 0.1 mM Na_2EDTA. Absorbance was measured at 260 nm.

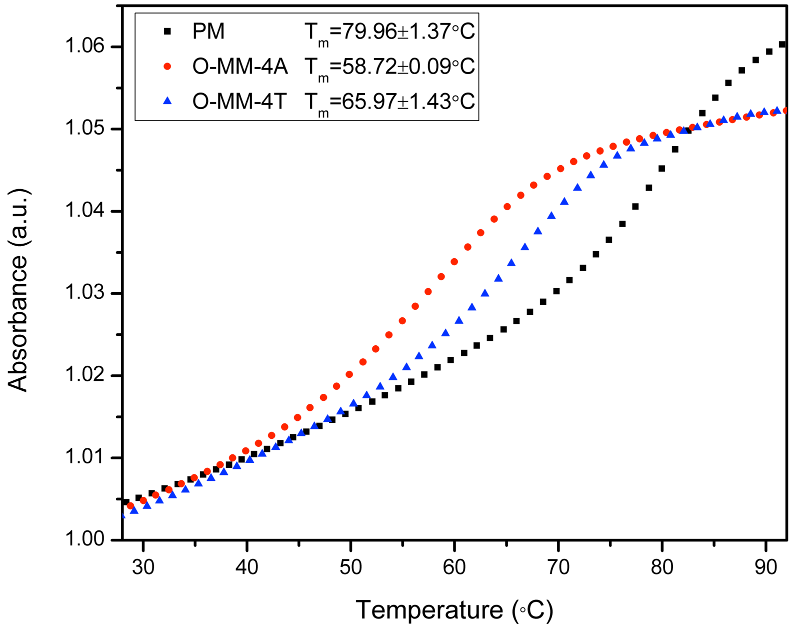

Figure S3. UV-vis melting analysis of struc_γPNA with PM, O-MM-4T and O-MM-4A DNA. All samples contained 2 μM DNA and 2 μM unstruc_γPNA in 100 mM NaCl, 10 mM Tris-HCL (pH = 7.4), and 0.1 mM Na_2EDTA. Absorbance was measured at 260 nm. Melting temperatures are higher and destabilizations smaller than for unstructured probe (Figure S2).
